# Nanoparticle Exposure and Hormetic Dose–Responses: An Update

**DOI:** 10.3390/ijms19030805

**Published:** 2018-03-10

**Authors:** Ivo Iavicoli, Veruscka Leso, Luca Fontana, Edward J. Calabrese

**Affiliations:** 1 Department of Public Health, University of Naples Federico II, 80131 Naples, Italy; ivo.iavicoli@unina.it (I.I.); veruscka.leso@gmail.com (V.L.); 2 Institute of Public Health, Catholic University of Sacred Heart, 00168 Rome, Italy; lfontana73@yahoo.it; 3 Department of Environmental Health Sciences, University of Massachusetts, Amherst, MA 01003, USA

**Keywords:** nanomaterial, dose–response relationship, hormesis, low doses

## Abstract

The concept of hormesis, as an adaptive response of biological systems to moderate environmental challenges, has raised considerable nano-toxicological interests in view of the rapid pace of production and application of even more innovative nanomaterials and the expected increasing likelihood of environmental and human exposure to low-dose concentrations. Therefore, the aim of this review is to provide an update of the current knowledge concerning the biphasic dose–responses induced by nanoparticle exposure. The evidence presented confirmed and extended our previous findings, showing that hormesis is a generalized adaptive response which may be further generalized to nanoscale xenobiotic challenges. Nanoparticle physico-chemical properties emerged as possible features affecting biphasic relationships, although the molecular mechanisms underlining such influences remain to be fully understood, especially in experimental settings resembling long-term and low-dose realistic environmental exposure scenarios. Further investigation is necessary to achieve helpful information for a suitable assessment of nanomaterial risks at the low-dose range for both the ecosystem function and the human health.

## 1. Introduction

**Figure 1:**
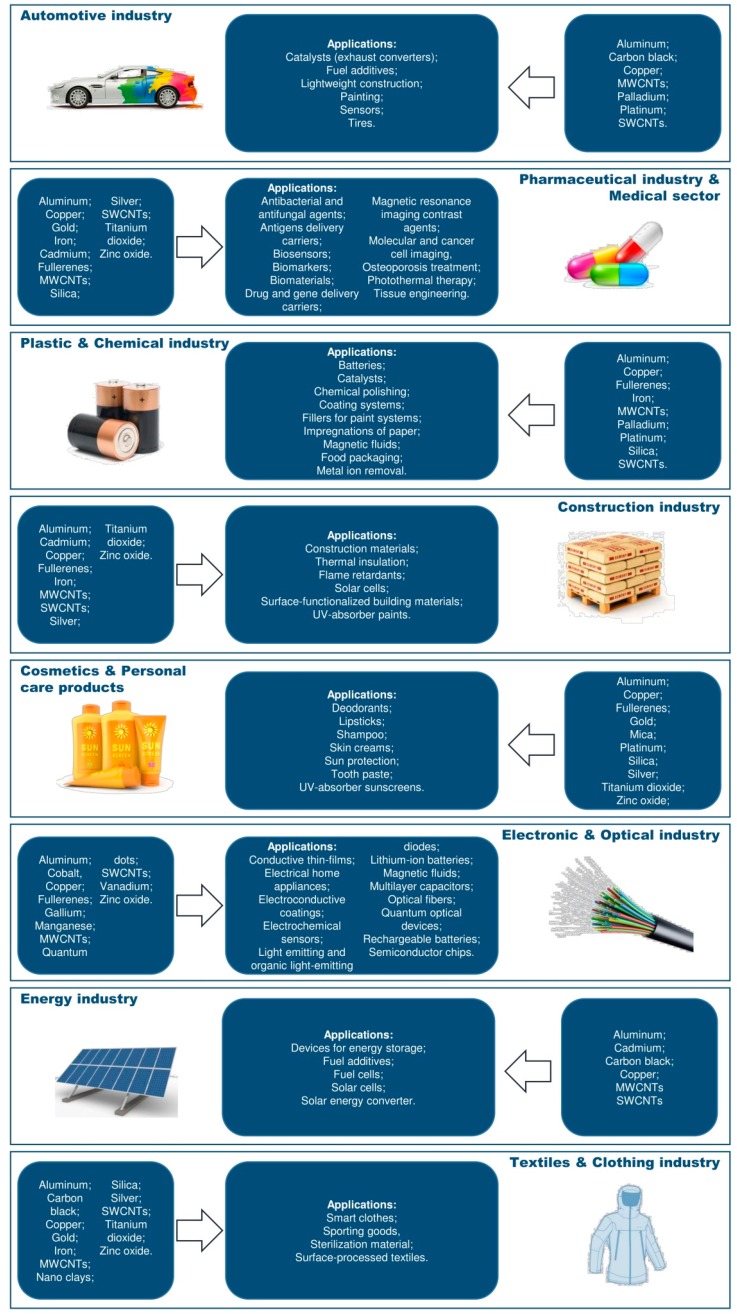
Main industrial uses and consumer product applications of engineered nanomaterials.

Rapid advances in technology have offered the opportunity to produce innovative engineered nanomaterials (ENMs), which find application in several production fields, including electrical engineering, chemistry, material sciences, biomedicine, textile, cosmetic and food industry [1] (Figure 1). Due to this widespread use, the possibility of environmental release of such chemicals, as well as the likelihood of human exposure in both community and occupational settings has notably increased [2,3]. Consequently, from a public health perspective, there is the need to assess ENM fate once they are released and dispersed into the environment, as well as to define their potential impact both on the ecosystem and human health.

In this regard, considering the relevance of real environmental low-dose exposure scenarios, the evaluation of the biological dose–response relationships induced by low concentrations of ENMs has emerged as an important issue for the nano-toxicological research. Within this low-dose exposure range, a great interest has been focused on assessing the prevalence of the hormetic phenomenon defined as a “biphasic dose–response relationship characterized by a low-dose stimulation and high-dose inhibition” [4]. Therefore, the concept of hormesis has generated considerable attention also in the area of nano-toxicology and nano-risk assessment [5]. In a recent assessment of nanoparticle toxicology, carried out by our group, we reported that different types of ENMs were able to induce a hormetic response in both in vitro and in vivo models involving broad biological and ecological domains [6,7,8]. The present paper extends these findings concerning the most recent evidence on ENM induced hormetic-like biphasic dose responses and their mechanistic foundations.

## 2. Results

### 2.1. In Vitro Studies

Recent in vitro experiments have demonstrated that hormetic dose–responses were induced by metal-based ENMs (Table 1). Among those, silver nanoparticles (Ag-NPs), commercialized in household antiseptic sprays, food packaging, clothes and antimicrobial coatings of medical devices [9,10,11], caused biphasic dose–responses in different cell lines, i.e., lung, gastrointestinal and hepatic cells [12,13,14]. Moreover, nano-diamonds (NDs) and silica nanoparticles (SiO_2_-NPs) employed as drug delivery systems, magnetic resonance imaging contrast enhancers and biosensors for intracellular pathogens induced hormetic responses in dermal fibroblasts [15].

#### 2.1.1. Lung Cells

The size, solubility and agglomeration state of Ag-NPs affected the hormetic adaptive response exerted in lung cells. The pre-treatment of human A549 lung epithelial cells with low doses of Ag-NPs reduced their toxic effects at higher concentrations, as well as the toxicity of acrolein, compared to non-pretreated cells directly exposed to the toxic challenge [12]. Interestingly, this effect was statistically significant only for colloidal Ag-NPs that were characterized by a smaller NP primary size, higher solubility and relatively lower agglomeration behavior.

#### 2.1.2. Gastrointestinal and Hepatic Cells

The hormetic response observed in HepG2 hepatoma cells [13] was related to the Ag-NP size even at non-cytotoxic concentrations. A hormetic cell proliferation response was also more evident with greater size NPs compared to smaller ones. Importantly, the Ag-NP induced cell proliferation was not dependent on Ag^+^ ion release, suggesting that the hormetic phenomenon is an intrinsic Ag-NP effect. Although not statistically significant, promotion of Caco2 gastrointestinal cell proliferation was induced by low Ag-NP concentrations, while a significant inhibition was evident at higher exposure treatments [14]. Using the same cell type, ZnO-NPs induced a significant dose-dependent cytotoxic action, pointing out the possible role of NP chemical composition in affecting biphasic responses in cellular viability.

#### 2.1.3. Dermal Cells

Differently shaped and chemically composed NPs such as NDs and SiO_2_-NPs affected an hormetic response in cellular proliferation of human skin fibroblasts exposed for a sub-acute period (48 h) [15]. A comparable stimulation of cell growth and a wound healing promoting effect was evident also for longer exposure periods to non-cytotoxic concentrations (up to eight days). These results imply that NDs and SiO_2_-NPs at low doses may exert mild stress-induced beneficial hormetic effects through improved survival, longevity, maintenance, repair and function of human cells which were not affected by the treatment length.

### 2.2. In Vivo Studies

Several biological models, from bacterial strains to rats including algae, plants, nematodes and superior aquatic organisms, were reported to respond to NP challenges, particularly to the Ag-NP insults, according to a hormetic dose–response, in a wide range of experimental conditions (Table 2). Such findings suggest that biphasic relationships may reflect an adaptive response of a broad range of in vivo models to ENMs.

#### 2.2.1. Bacteria Strains

The anti-bacterial properties of Ag-NPs under sub-lethal concentrations remains to be further explored [40]. Neither different NP size nor diverse surface functionalization was reported to affect the hormetic response induced on *Escherichia coli* strain by polyethylene glycol (PEG)- or polyvinyl-pyrrolidone (PVP)-coated Ag-NPs [16]. Ag^+^ ions released from Ag-NPs were hypothesized as possible triggers of the hormetic response.

With regard to the NP effects on marine bacteria, a species specific biphasic response concerning the O_2_ uptake rate was reported by Echavarri-Bravo et al. [18] on pure bacterial cultures. Gram-positive bacteria showed an enhanced respiratory activity at low concentrations of NM-300 and Ag-NPs contained in a household product (Mesosilver) and a reduction at higher concentrations. In contrast, no hormetic effect was detected for the Gram-negative bacterial strain investigated. Furthermore, no biphasic dose–response was induced by Ag-NP exposure in the respiratory activity of a mixed population of natural bacteria cultured under environmentally relevant conditions. 

NP induced hormesis can be also affected by the presence of other compounds, such as amino-acids in culture media [17]. In fact, an increase in bacterial viability was observed at higher concentrations when PVP-coated-Ag-NPs were applied together with cysteine compared to NPs alone. Therefore, these results suggest that cysteine affects a concentration dependent biphasic dose–response regulating the final Ag^+^ ion concentrations released from Ag-NPs.

#### 2.2.2. Environmental Microbial Communities

The induction of hormetic dose–response effects on microbial ecosystem functions was investigated by Yang et al. [19] on three models of nitrogen-cycling bacteria exposed to sub-lethal concentrations of Ag-NPs. These xenobiotics induced an hormetic response in microbial ecosystems in a species-specific manner. In fact, nitrifying genes in *N. europaea* were significantly up-regulated at doses corresponding to the 10% of the silver nitrate (AgNO_3_) minimum inhibitory concentrations, while such transcriptional stimulatory effect was not more at higher concentrations. On the contrary, such relationships were not observed in *Pseudomonas stutzeri* and in *A. vinelandii* bacteria investigating the expression of the denitrifying or nitrogen-fixing genes. In a subsequent study, Yang and Alvarez [20] reported that sub-lethal concentrations of PVP-coated Ag-NPs promoted the development of biofilm growth in a mixed culture from a wastewater treatment plant. Regarding the possible molecular mechanisms underlying such effect, transcriptomic responses to PVP-coated Ag-NPs in *P. aeruginosa* showed a significant up-regulation of bacterial genes associated with biofilm formation as well as an increase in the level of production of sugar and protein components of the biofilm matrix. However, it remains to be elucidated what the potential public health risk may be from the presence of Ag-NPs in wastewater which could accelerate bio-fouling, bio-corrosion and harbor pathogenic bacteria proliferation.

As a confirmation that Ag-NPs may cause intracellular hormetic metabolic alterations, Zheng et al. [21] demonstrated a significant biphasic action of PVP-coated Ag-NPs on the nitrous oxide (N_2_O) production of nitrifying bacterial communities from the sediments of the Yangtze estuary. The stimulation of N_2_O production might be considered as a NP induced stress-response in nitrifying communities. This finding is globally significant as environmental inputs of Ag-NPs have been significantly increasing due to their rising usage and disposal levels worldwide [21,41,42,43]. In this context, Sheng et al. [22] evaluated whether the hormetic model could be applied to the effects of fresh and aged Ag-NPs on microbial communities in biological wastewater treatment systems (i.e., biofilm/activated sludge, planktonic mixed culture and pure culture of single strains). When low doses of such Ag-NPs were applied in activated sludge bioreactors fed with synthetic municipal wastewater, no significant effects could be observed on pollutant removal, while freshly prepared Ag-NPs could especially contribute to maintain the microbial community diversity in the activated sludge. This underlines the importance of assessing not only the toxicological behavior of differently sized, shaped or functionalized NPs, but also possible differences due to freshly prepared or aged ENMs which may demonstrate peculiar biological reactivity and/or modes of action. Overall, these results may be important, since a greater diversity of the microbial community of ten yield microbes more resistant to stress [44].

#### 2.2.3. Algae

Copper nanoparticles (Cu-NPs) have found application in a large range of production fields, including printing, lithium ion battery production, and biomaterial synthesis and are potentially released from industrial sewage into the aquatic environment [45]. Concerning possible hormetic effects on aquatic ecosystems, the exposure of marine diatom *P. tricornutum* to low concentrations of Cu-NPs showed a slight growth stimulation, while the treatment with higher concentrations induced a significant inhibition of the growth rate [23]. However, these results are quite different compared to those previously obtained by Morelli et al. [46] who demonstrated that CdSe/ZnS quantum dots did not exert a biphasic response in the same marine diatom. These contrasting findings would suggest that the hormetic adaptive response may be significantly affected also by the ENMs chemical composition.

The hormetic phenomenon described by Zhu et al. [23] was also confirmed by the results obtained with Cu nano-aquachelates carboxylated with citric acid on the biomass accumulation of the green algae *Chlorella vulgaris* [24]. This latter finding may be dependent on the increase in inorganic copper concentration which may impair biochemical and physiological processes in algal cells. Therefore, the hormetic response may be a slight overcompensation following an initial disruption in homeostasis induced by the xenobiotic insult [5]. Once again, the lack of a hormetic response following treatment with Selenium-NPs carboxylated with citric acid in the same algal model supported the key action of NP chemical composition in determining such phenomenon.

#### 2.2.4. Plants

As regards the Ag-NP interactions with plants, a size-dependent hormetic effect was observed in poplars (*Populus deltoides × nigra*) and *Arabidopsis thaliana* hydroponically exposed to PEG-coated 5 and 10 nm Ag-NPs, and carbon-coated 25 nm Ag-NPs at a wide range of concentrations [26]. In fact, although a biphasic response was reported for both the investigated materials, a lower stimulatory concentration was determined for 5 or 10 nm Ag-NPs compared to 25 nm Ag-NPs. This result could be consequent to the easier Ag accumulation, as well as to the faster dissolution of smaller NPs. Nevertheless, the different surface functionalization of these NPs does not allow to exclude a possible affecting role of such parameter in determining different dose–response relationships.

To investigate the effects of Ag-NPs on plant development, regenerated shoots of *Vanilla planifolia* were exposed to five different concentrations of Ag-NPs using a temporary immersion bioreactor system [28]. A significant stimulation of shoot multiplication and elongation was induced by the lowest doses of exposure, while the highest one significantly inhibited both processes. Ag-NPs, at low doses, may act by improving nutrient uptake efficiency in plants [30,47], as well as activating anti-oxidant defense mechanisms which may contribute to the metal induced hormesis in plants [48]. High doses of Ag-NPs, however, may block nutrient transportation by ionic channel competition and, reducing shoot number and length, induce phytotoxicity. Although achieved with differently functionalized NPs and in diverse plant models, Bello-Bello et al. [29] reported comparable results with Sugarcane shoots treated with PVP-coated Ag-NPs under similar experimental conditions.

These findings are in good agreement with those previously obtained by Jhanzab et al. [30], Razzaq et al. [31] and Salama et al. [27] in wheat seedlings, common bean and corn plantlets, although these studies considered different plant growth parameters (i.e., yield attributes and nitrogen (N)-phosphorous (P)-potassium (K)-nutrient use [30]; leaf area, root biomass, fresh weight and dry weight [31]; and shoot and root length, chlorophyll, carbohydrate and protein content [27]). Interestingly, in the study by Razzaq et al. [31], not only the concentration, but also the duration of exposure seemed to influence the seedling hormetic growth response. Indeed, increasing concentrations and exposure time were reported to induce a decrease in leaf area, fresh weight and dry weight.

As a further consideration, nanotechnology has the potential to make an impact on several agricultural challenges including the improvement and protection of agronomic yields and crop production [49]. However, a responsible development of such application requires a deeper knowledge of the toxicological behavior of potentially employed ENMs on ecosystems and in particular on the modes though which they might influence the physiology and development of plants as well as dose–response relationships. In this regard, Tiwari et al. [25] demonstrated that low concentrations of multi walled carbon nanotubes enhanced the germinative growth of maize seedlings, while higher ones induced a significant inhibition. A biphasic dose–response trend was also observed in the modulation of the oxidative stress process in soybean seeds treated with different concentrations of a colloidal solution (containing several nano-metals) used for plant nutrition and physiological regulation [32]. This colloidal solution may act as stress factor able to induce non-linear adaptive responses. Overall, these results appear absolutely important considering the possible future widespread ENM application into the agricultural practice.

#### 2.2.5. Nematodes and Larvae

A key question in hormesis research has been how observation of low dose stimulation for a single trait in short term studies are related to effects on multiple traits over the full life time exposure. In this regard, Tyne et al. [33] studied the hormetic phenomenon focusing on full life-cycle responses of nematode *Caenorhabditis elegans* to Ag-NPs. Interestingly, while a biphasic response in the reproduction rate was evident following a sub-acute period of exposure, this phenomenon was no longer observable during a full life-cycle extended exposure period. This suggests the importance to investigate the hormetic phenomenon specifically focusing in a life-cycle perspective which may provide a more suitable analysis of the putative biphasic dose–response.

An original approach to assess the effects of NPs on the ecosystem functions was used by Bour et al. [34] investigating in benthic invertebrate *Chironomus riparius* larvae the teratogenic effects of three different types of CeO_2_-NPs. Spherical and smaller, -tri ammonium citrate coated CeO_2_-NPs, reduced both the frequency and seriousness of mouthparts deformities in larvae, therefore exerting a protective action on teratogenic effects compared to controls, as well as to the other two types of CeO_2_-NPs. Overall, this supports the importance to assess the role of NP physico-chemical features, including size, shape and surface functionalization in determining a biphasic dose–response trend.

#### 2.2.6. Superior Aquatic Organisms

Metal oxide ENMs, such as Zinc oxide (ZnO-NPs) and CuO-NPs, have found considerable application in a variety of consumer products, including cosmetics, and antimicrobial products such as textile fibers, wood preservatives and antifouling coatings [35,36,37]. Therefore, aquatic ecosystems may be contaminated by such ENMs that may be released into the environment through waste water discharges and wash off during recreational activities.

In several studies addressing the hormetic phenomenon in amphibian models, the different time periods of exposure, adopted in the experimental settings were able to affect hormetic manifestation [35,36,37]. Concerning the sub-acute effects of ZnO-NPs on amphibian growth endpoints, a non-linear relationship was reported for growth throughout metamorphosis [35] and tadpole lengths [36]. When the effects of CuO-NPs were assessed on amphibian growth, no effects were acutely evident [36], while in a sub-chronic time frame, all tested concentrations resulted in embryos mortality [37]. In contrast, the chronic application of low doses of these NPs demonstrated a hormetic effect, stimulating tadpole metamorphosis and growth, while higher ones elicited significant mortality. Therefore, these results suggest the importance of considering both the NP chemical composition and the exposure length when assessing non-linear dose–responses.

This latter aspect was also confirmed by Saggese et al. [38] treating Red Sea mussel *B. pharaonis* with Ag-NPs. In this study, the lowest and highest doses of exposure could both increase the respiration rate of mussels compared to the controls and the middle dose groups. However, temporal analysis of the dynamics of such parameter during the eight days of exposure supports different compensation strategies to the diverse applied concentrations. In fact, the lowest exposure dose showed a delayed intensification of the respiration rate at the last day of treatment, while the highest concentration induced an increase in the first two days followed by a return to control values in the last period of exposure. This overall response may suggest that multiple factors, other than the nominal dose may play a role in affecting the non-linear dose–response to the NM insults.

#### 2.2.7. Rats

Recently, our research group, investigating the possible toxic effects of palladium NPs (Pd-NPs) on the immune system of female Wistar rats, observed an interesting biphasic dose–response correlated to the production and release of different cytokines [39]. In detail, the intravenous exposure of these laboratory animals to 0.012, 0.12, 1.2 and 12 μg/kg of Pd-NPs caused a slight decrease at the lowest exposure dose and an increase thereafter with increasing doses. These findings would suggest that the initial decrease in cytokine levels could represent an adaptive compensatory response of the immune system induced by a disruption in homeostasis following the Pd-NP exposure.

Pd-NPs have a very high catalytic activity due to their high surface area to volume ratio and high surface energy and, for this reason, their catalyst potential is currently exploited in a wide range of chemical applications. Moreover, Pd-NP applications are extensively used in electronics, automotive and medicine.

## 3. Discussion

In recent years, the knowledge concerning the hormetic dose–response induced by ENMs has been rapidly growing and appeared relevant for the safe application of such innovative materials as well as to understand the health impact they may have for the general and occupational exposed populations.

As stated in our previous assessment [7], this update confirms that different types of ENMs such as metal and metal oxide NPs, SiO_2_-NPs and carbon-based ENMs induced an hormetic response in both in vitro and in vivo experiments. Although specific cellular, plant, microbial, invertebrate and animal models were investigated, the “generalized” hormetic response we found among different biological systems supports hormesis as an evolutionary-based adaptive response across all forms of investigated life [50].

**Figure 2:**
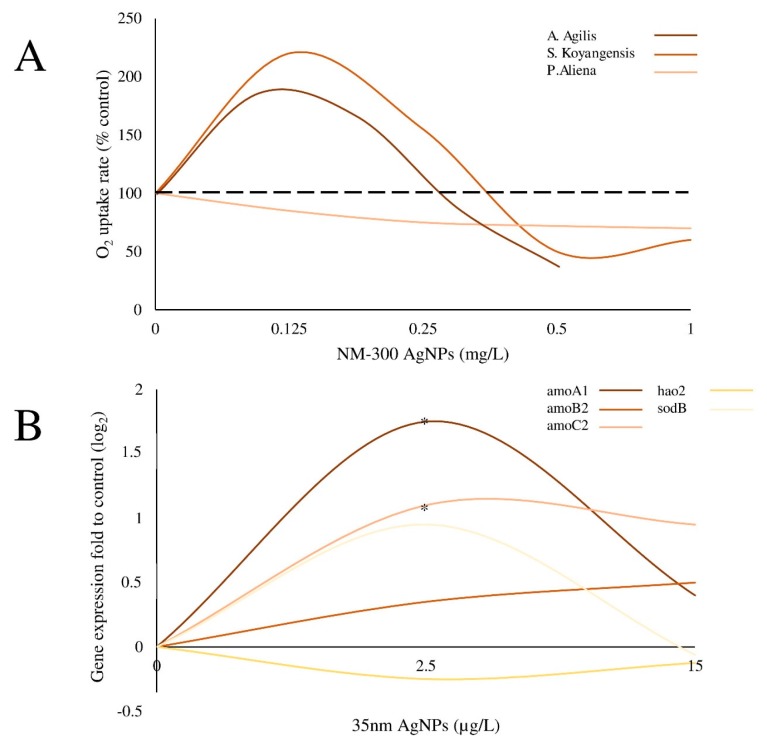
Examples of species-specific biphasic response observed in different bacteria. (**A**) The respirometry assays showed that NM-300 AgNPs were able to induce a hormetic response only in Gram-positive bacteria (*A. agilis* and *S. koyangensis*) (modified by Echavarri-Bravo et al. [18]). (**B**) The exposure of *N. europaea* to 35 nm AgNPs affected the transcriptional activity of nitrifying genes (*amoA1*, *amoB2*, *amoC2*, *hao2* and *sodB*) showing a biphasic response. This effect was not evident in other bacteria (*P. tutzeri* and *A. vinelandii*) (modified by Yang et al. [19]). * Indicates statistical significance (*p* < 0.05).

However, future research in this sense, should be aimed to verify such finding in a greater number of biological models in order also to define possible differences in biphasic responses potentially related to the intrinsic properties of the addressed systems. In some cases, in fact, a species-specific susceptibility to the hormetic phenomenon was reported [18,19] (Figure 2), suggesting the need to define the interaction between NPs and the diverse biological systems as well as the molecular processes underling non-linear toxicological relationships. In this regard, the limited number of animal investigations claims for additional in vivo studies aimed to understand biphasic reactions in more advanced biological entities.

Concerning the diverse end-points investigated, a “generalized” biphasic response could be also detected. A wide range of parameters, including cellular and bacterial viability; bacterial gene expression regulation; algal and plant indices of germination, growth, photosynthetic capability, and oxidative stress; as well as invertebrate organism development parameters showed a significant stimulation at the lower concentrations of treatment, while opposite trends were observed at higher levels of exposure. In NP exposed animals, a significant inhibition of cytokine production was evident at the lowest dose of treatment while a significant stimulation was induced by higher doses.

However, since most reviewed investigations were focused on Ag-NPs, in relation to their greater application in consumer products, need for future research focused on a broader range of ENMs to define possible common modes of action as well as peculiarities due to the specific NP physico-chemical properties.

**Figure 3:**
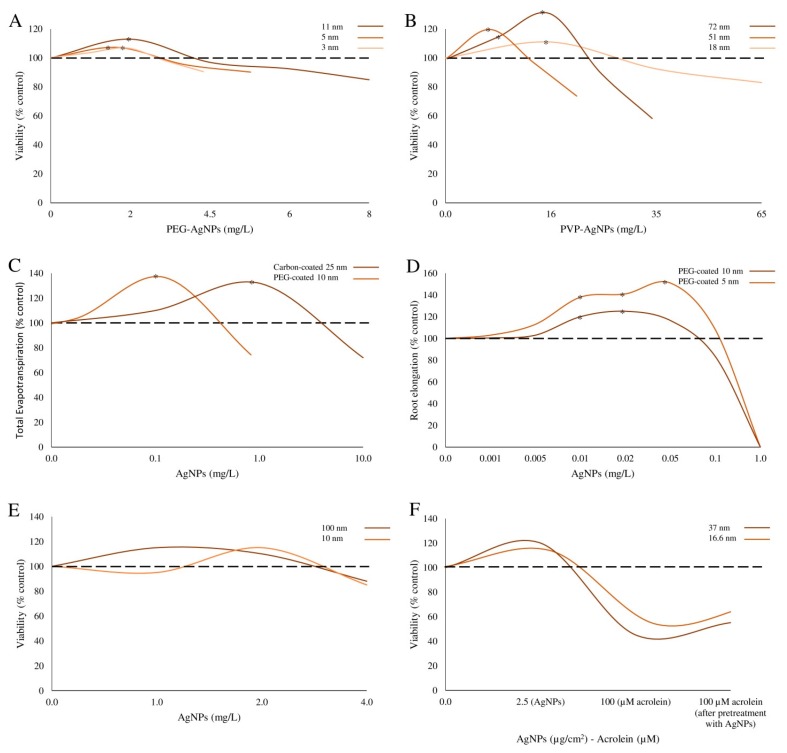
Examples of NP size-dependent biphasic response. (**A**,**B**) In *E. coli* cells, the exposure to Ag-NPs induced a hormetic response regardless of NP size and functionalization (modified by Xiu et al. [16]). (**C**) Biphasic response of cumulative evapotranspiration was observed in poplars (*Populus deltoides × nigra*) exposed to carbon- and PEG-coated Ag-NPs of 25 and 10 nm, respectively (modified by Wang et al. [26]). (**D**) PEG-coated Ag-NPs of 5 and 10 nm affected the root elongation of *Arabidopsis thaliana* showing a hormetic effect (modified by Wang et al. [26]). (**E**) Ag-NPs of different size (10 and 100 nm) showed a hormetic cell proliferation response in HepG2 cells (modified by Jiao et al. [13]). (**F**) Pretreatment of human A549 lung epithelial cells with low doses of Ag-NPs reduced the cytotoxic effect caused by acrolein and/or by exposure to higher doses of the same NPs (modified by Sthijns et al. [12]). * Indicates statistical significance (*p* < 0.05).

**Figure 4:**
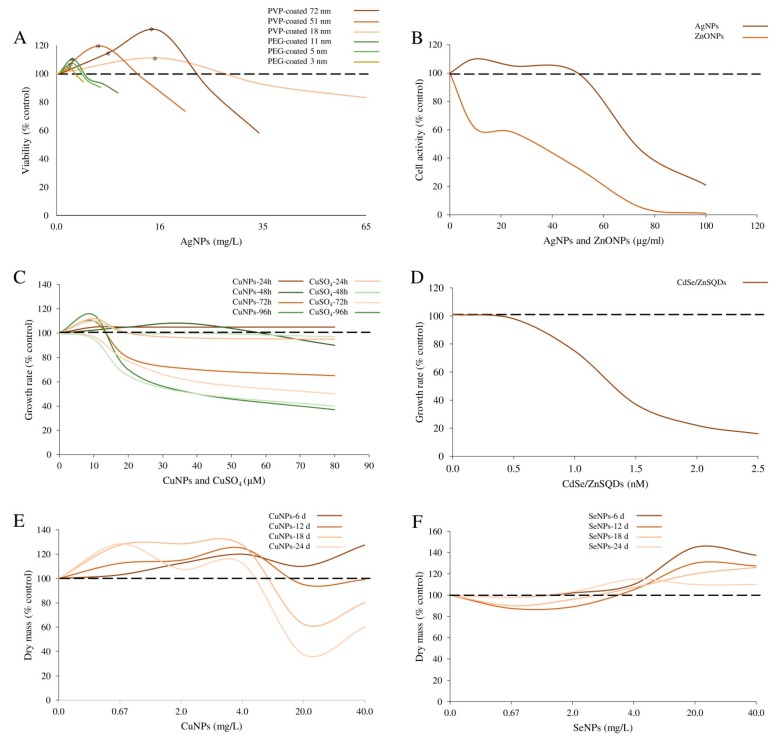
Influence of NP chemical composition on the induction of biphasic response. (**A**) In *E. coli* cells, the exposure both to PVP- and PEG-coated Ag-NPs induced a hormetic response (modified by Xiu et al. [16]). (**B**) In Caco2 cells exposed for 36 h to different concentrations of Ag-NPs and ZnO-NPs, the hormetic response was induced only by Ag-NPs (modified by Kang et al. [14]). (**C**,**D**) Cu-NPs at higher concentrations inhibited *P. tricornutum* growth, while at lower concentrations slightly stimulated growth (modified by Zhou et al. [23]). In the same experimental model, increasing concentrations of CdSe/ZnS-QDs did not induced a hormetic response (modified by Morelli et al. [46]). (**E**,**F**) Cu-NPs and Se-NPs induced a different kind of biphasic response related to the grown of *Chlorella vulgaris* (modified by Mykhaylenko and Zolotareva, [24]). * Indicates statistical significance (*p* < 0.05).

In this regard, conflicting results have been reported on the potential role of NP size in influencing the hormetic response in different biological systems (Figure 3). For example, stimulatory action of Ag-NPs was greater for NP formulations with a smaller diameter in human cell [12], plant [26] and larval models [34], than for NPs with a greater size [13]. Therefore, it can be speculated that factors other than size, including the original state of the material, e.g., powder vs. colloidal dispersion [12], the NP chemical composition [14,16,23,24] (Figure 4), the agglomeration status [12], and ENM surface functionalization [13,16,26] (Figure 4), should be considered as possible key factors in the hormetic response to NP insults.

**Figure 5:**
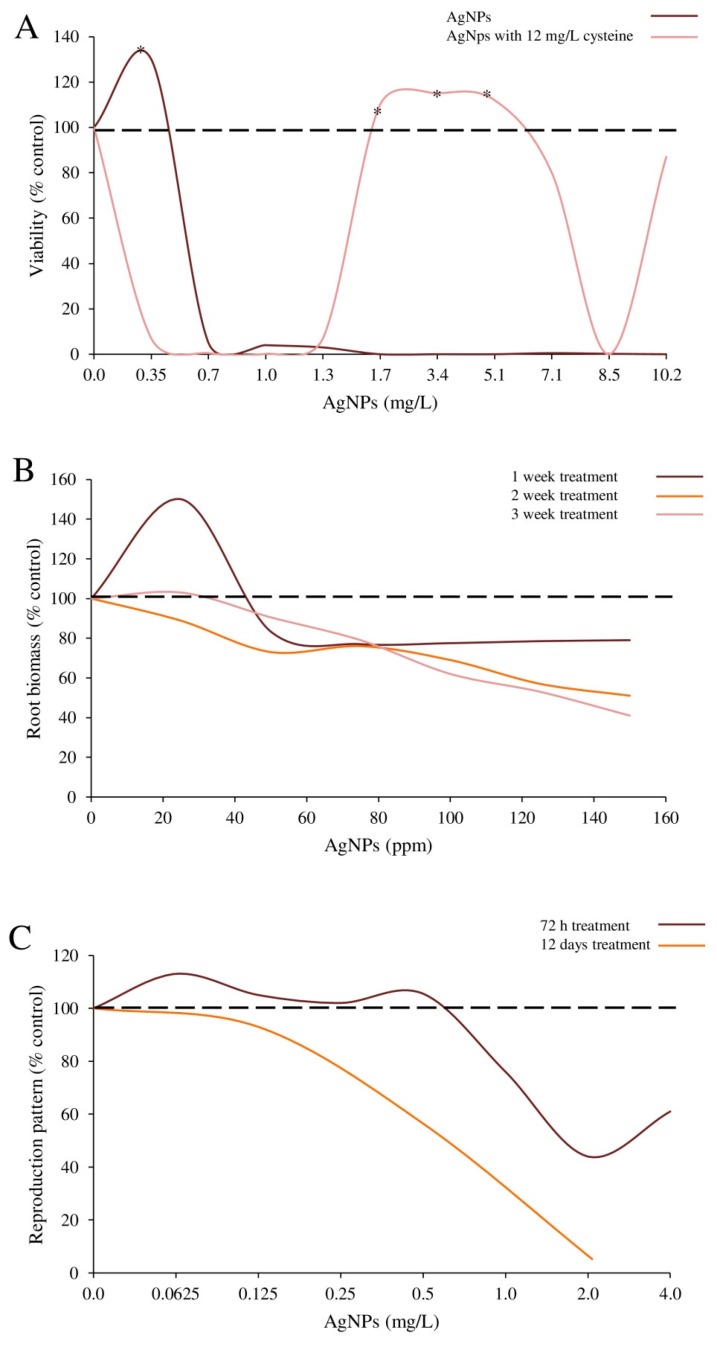
Influence of experimental conditions on the occurrence of hormesis. (**A**) The addiction of cysteine in culture media significantly affected the hormetic behavior of Ag-NPs (modified by Guo et al. [17]). (**B**) Duration of exposure might influence the induction of a biphasic response by Ag-NPs (modified by Razzaq et al. [31]). (**C**) The hormesis response induced by Ag-NPs in *Caenorhabditis elegans* is evident following a sub-acute period of exposure, while after 12 days it was no longer observable (modified by Tyne et al. [33]). * Indicates statistical significance (*p* < 0.05).

However, as a follow up point of discussion, not only the intrinsic, primary NP properties should be considered, but also the features secondarily acquired through the interactions with the chemical experimental settings. In this regard, differences between pure bacteria cultures and microbial communities explored under conditions mimicking natural ecosystems, suggest the need to address the hormetic phenomenon in suitable environmentally relevant exposure settings [18]. The stability of NPs in natural or waste waters, in fact, may be related to the reciprocal interactions between their primary physico-chemical properties and environmental factors, i.e., the salinity, the presence of natural organic matter, inorganic ligands which may all contribute to modify the biological identity and bioavailability of NPs and their consequent toxicological behavior [21]. This challenging aspect should be carefully considered when evaluating the NP impact on bacterial community functions, essential for nutrient cycling and bioremediation, under realistic exposure settings. This issue also underlines the importance to assess the impact of the co-exposure to other substances in affecting NP hormetic responses [17] (Figure 5).

In line with these ideas, the role of physico-chemical properties, acquired by NPs when interacting with cell growth media or biological fluids, in affecting hormetic responses, should be elucidated in future investigations. Importantly, culture media, as well as physiological compartments, possess their own set of proteins that may be adsorbed on NPs when they interact with different physiological environments. This produces the so-called bio-molecular coronas that may change the biological identity of ENMs, supporting the need to further assess, in vivo, the contribution of diverse routes of exposure in affecting biphasic reactions.

Extensive evidence has reported that preconditioning and adaptive responses are manifestations of hormetic relationship [51,52]. “Preconditioning” is a term used in biology and physiology to describe a process where small doses of potential hazardous/stressors evoke an adaptation and increased resistance to the noxious insult [53]. In line with this concept, a major finding by Sthijns et al. [12] indicated that the low-dose Ag-NP pretreatment protected against the adverse effects of a toxic concentration of another substance. This finding implicates a hormetic effect induced by a compound different than the chemical of exposure. Additionally, to better understand preconditioning processes, it is necessary to better define to what extent challenges, in repeated dose rates, would influence the hormetic effects. These findings may guide a suitable risk assessment process, particularly for consumers and workers who are expected to be repeatedly and chronically exposed to NPs. In line with this idea, the hormetic phenomenon should be verified not only through short-time periods of exposure, but also during chronic exposures [33] (Figure 5).

The precise molecular mechanisms of hormesis induced by NPs are still unknown. Additionally, it is controversial whether hormesis may be a nano-specific phenomenon or a dose–response relationship dependent on the NP ion release [16,23]. In this regard, as the existing studies used variable types of NPs and conditions of exposure, more systematic experiments are needed to elucidate the signaling pathways activated by NPs. In view of the established importance of oxidative stress and redox-signaling in the toxicity of many ENMs, it will be necessary to also investigate whether such processes may mediate hormetic adaptation [28].

## 4. Materials and Methods

A systematic search/review was conducted in accordance with the Preferred Reporting Items for Systematic Reviews and Meta-Analyses (PRISMA) Statement criteria [54]. PubMed, Scopus and Web of Science databases were searched to identify papers addressing the hormetic dose–response phenomenon in response to ENM exposure in different biological systems. The initial search took place in September 2017. The above-mentioned databases were last consulted on 29 January 2018.

We carried out a preliminary systematic search for the terms “nanoparticles” (NPs) to assess the exposure, and “hormesis” to identify the outcome of the research. This preliminary search retrieved 31, 25 and 38 references through Scopus, PubMed and Web of Science, respectively. To include a broader range of published papers, we extended our research strategies to the terms “NPs” and “biphasic dose response” which retrieved a total of 29, 17, and 14 references for the three searched databases, respectively. Additionally, an ulterior search was performed with the string “NPs” and “non-linear dose response”, that found a total of 16 articles in Scopus, 9 in PubMed and 9 in Web of Science databases.

Titles and abstracts of identified studies were examined applying the following predefined eligibility criteria:

- In vitro and in vivo and in field experimental studies published in peer-reviewed scientific journals;
- Published in English; and
- Exploring the hormetic biphasic dose–response relationship induced by nanoparticle exposure.

Exclusion criteria were applied to studies not satisfying the aforementioned selection criteria or included in our previously published literature reviews [5,7].

**Figure 6:**
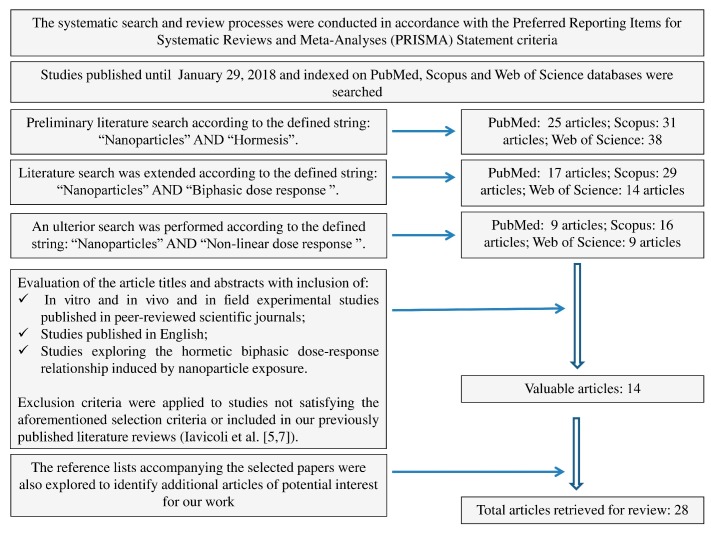
Flow diagram of literature search.

After the exclusion of those articles that did not met the inclusion criteria and removal of duplicates, only 14 were considered suitable for our scope by title and abstract screening. All full texts of the papers considered valuable for the aim of our review were obtained and a critical evaluation was performed. The reference lists accompanying the selected papers were also explored to identify additional articles of potential interest for our work (Figure 6). Overall, our search retrieved a total of 28 publications for review. 

## 5. Conclusions

This review provides an update of the available knowledge concerning the hormetic-like biphasic dose–responses induced by NP exposures. Biphasic dose–responses have been observed in a wide range of biological in vitro and in vivo models, supporting hormesis as a generalized adaptive response to nanoscale xenobiotic challenges. Different NP physico-chemical characteristics were suggested as playing a key role in determining such dose–response relationship, although further research is necessary to define molecular mechanisms underlining possible different results. Additionally, investigations should be focused to clarify those factors determining possible variabilities in NP hormetic responses due to the characteristics of the biological systems investigated and experimental conditions adopted, e.g., longer exposures, environmentally relevant conditions of treatment and the presence of other co-exposed substances. Overall, this may provide helpful information to deeper understand the mechanistic foundation of the NP induced hormetic responses and achieve an adequate assessment of ENM risks at the low-dose range for both the ecosystem and human health.

## Figures and Tables

**Table 1:** In vitro studies demonstrating the presence of a nanoparticle induced hormetic dose–response.

Type of Nanoparticles	NP-Physicochemical Characterization	Cell Line Investigated	Experimental Design	Assessed Endpoint	Results	References
Ag-NPs	Shape: spherical; Size: 10 and 100 nm; Hydrodynamic diameter: 19.6 ± 0.5, 99.5 ± 0.3 nm.	HepG2	Cells were exposed to 1 and 2 μg/mL NPs for up to 72 h.	Cell viability	Cell viability increased to ~125% and ~150% relative to controls with 10 and 100 nm Ag-NPs after 48 and 72 h treatments with both exposure concentrations.	Jiao et al. [13]
Ag-NPs ZnO-NPs	Size: 30 nm.	Caco2	Cells were exposed to 0–100 μg/mL NPs for up to 36 h.	Cell viability	Cell viability was slightly (n.s.) increased by 10, 25, 50 μg/mL Ag-NPs for 36 h, and was reduced by higher doses at all 3 time points. ZnO-NPs induced a dose-dependent decrease in cell viability at all 3 time points.	Kang et al. [14]
Colloidal (c-) and powder (p-) Ag-NPs	Shape: near-spherical to elongated (p-Ag-NPs); spherical c-Ag-NPs; Size: 37.0 ± 13.0 nm (p-Ag-NPs); 16.6 ± 4.4 nm (c-Ag-NPs); Hydrodynamic diameter: 690.6 ± 74.5 (p-Ag-NPs); 242.5 ± 93.5 nm (c-Ag-NPs).	A549	Cells were exposed to c-Ag-NPs (5 μg/cm^2^) or p-Ag-NPs (2.5 μg/cm^2^) for 24 h followed by 80 or 60 μg/cm^2^ NPs, respectively, or acrolein as an environmental pollutant (100 μM).	Cell viability	Cell viability increased in cells pre-treated with low concentrations of NPs (2.5 and 5 μg/cm^2^), compared to cells directly exposed to the toxic challenge.	Sthijns et al. [12]
NDs and SiO_2_-NPs	Size: <10 nm (NDs); 12 nm SiO_2_-NPs.	FSF1	Cells were exposed to 0–100 μg/mL NPs for 48 h.	Cell viability	Cell viability was significantly increased by 0.5–2.5 μg/mL SiO_2_-NPs and 0.5 μg/mL NDs. Above 5 μg/mL there was a progressive decrease.	Mytych et al. [15]

A549, human lung adenocarcinoma cells; Ag-NPs, Silver nanoparticles; Caco2, human epithelial colorectal adenocarcinoma cells; FSF1, Normal diploid human facial skin fibroblasts; HepG2, human hepatoma derived cell line; n.s., not significant; NDs, nanodiamonds; SiO_2_-NPs, silica nanoparticles.

**Table 2:** In vivo studies demonstrating the presence of a nanoparticle induced hormetic dose–response.

Type of Nanoparticles	NP-Physicochemical Characterization	In Vivo Model	Experimental Design	Assessed Endpoint	Results	References
**Bacteria strains**						
PEG- and PVP-coated Ag-NPs	PEG-Ag-NPs Shape: spherical; Size: 2.8 ± 0.47; 4.7 ± 0.20; 10.5 ± 0.59 nm. PVP-Ag-NPs Size: 20, 40 and 80 nm.	*E. coli* strain K12 (ATCC 25404)	Bacteria were exposed to 0–65 μg/mL NPs for 6 h.	Bacteria viability	PEG-Ag-NPs: higher survival rates, 6%, 7% and 13% were induced by 2.8, 4.7 and 10.5 nm NPs at 2.2, 1.8 and 2.0 μg/mL, respectively. PVP-Ag-NPs: higher survival rates, 11%, 17%, and 21% were induced by 20, 40 and 80 nm NPs at 16.4, 5.7 and 6.7 μg/mL, respectively.	Xiu et al. [16]
PVP coated-Ag-NPs	Shape: spherical; Size: 7.59 ± 2.92 nm; Hydrodynamic diameter: 27.1 ± 2.2 nm.	*E. coli* strain ATCC 25922	Bacteria were exposed to 0.34–5.1 μg/mL NPs with or without cysteine (12.5 μg/mL).	Bacterial viability	Viability (without cysteine): 29.9% increase in bacteria treated with 0.34 μg/mL NPs compared to controls. Viability (with cysteine): stimulated by 1.7–5.1 μg/mL NPs.	Guo et al. [17]
NM-300 Ag-NPs; Mesosilver containing Ag-NPs	Size: 18.2 ± 7.3 and 14.0 ± 6.9 nm for NM-300 and Mesosilver, respectively.	Gram-positive bacteria *A. agilis* and *S. koyangensis* and the Gram-negative bacterium *P. aliena*	Bacteria were exposed to 0–1 μg/mL Ag-NPs (NM- 300) and Ag-NPs in a household product. Responses could be detected within 0.5–1 h.	O_2_ uptake	Gram-negative strain *P. aliena*: reduced O_2_ uptake rate in a concentration dependent manner Gram-positive strains (*A. agilis* and *S. koyangesis*): increased uptake rate at low concentrations (<0.25 μg/mL) compared to controls; reduced uptake at ≥0.5 μg/mL.	Echarri-Bravo et al. [18]
**Environmental microbial communities**						
Ag-NPs	Size: 35 nm; Hydrodynamic diameter: 35.4 ± 5.1 nm Surface functionalization: amorphous carbon coating.	*Pseudomonas stutzeri*, *Azotobacter vinelandii*, and *Nitrosomonas europaea*	*N. europaea*, *P. stutzeri* and *A. vinelandii* were exposed to 2.5, 20 and 25 μg/L NPs (10% of the AgNO_3_-MIC), respectively. *N. europaea* was also exposed to 15 μg/L NPs.	Transcriptional response to Ag-NP exposure	Ag-NPs had no significant effect on *P. stutzeri*, and *A. vinelandii* expression of denitrifying genes or nitrogen-fixing genes. In *N. europaea*, Ag-NPs (2.5 μg/mL) up-regulated the ammonia mono-oxygenase genes, while such effect was not evident at higher concentrations (15 μg/mL, 60% of the Ag + MIC).	Yang et al. [19]
PVP-coated Ag-NPs	Size: 10 nm.	Mixed culture of the effluent from West University Place wastewater treatment plant	Mixed and *P. aeruginosa* pure cultures were exposed to 45.7; 21.6 and 21.6 μg/L of PVP, Ag^+^, and NPs for 24 h, respectively.	Biofilm formation	The biofilm coverage fractions were 0.9 ± 0.2% for control, 0.9 ± 0.3% for PVP, 1.1 ± 0.3% for Ag^+^, and 5.2 ± 2.1% for PVP-Ag-NPs in the mixed culture. Biofilm development was increased by PVP-coated Ag-NPs in the *P. aeruginosa* pure culture.	Yang and Alvarez [20]
PVP-coated Ag-NPs	Size: 10, 30 and 100 nm.	Sediment slurry of the Yangtze Estuary	Sediment slurry was exposed to NPs 0–10,000 μg/L for 30 h.	N_2_O production	Nitrifier N_2_O production to Ag-NPs exhibited low-dose stimulation (<534, 1476, and 2473 μg/L for 10-, 30-, and 100-nm Ag-NPs, respectively) and higher dose inhibition.	Zheng et al. [21]
Fresh and aged Ag-NPs	Size: 74.2 ± 5.1 (aged NPs), 67.9 ± 1.0 nm (fresh NPs).	Sequencing batch reactors (*n* = 4) operating for over 3 months	Ag-NPs were added, 27 days after start-up, at a concentration of 1 mg Ag/L in influent (0.5 mg/L in the reactor) for over 2 months.	Microbial community analysis	Microbial analysis (16S gene-based sequencing): fresh Ag-NPs had the highest number of genes detected and richness.	Sheng et al. [22]
**Algae**						
Cu-NPs	Size: 10–30 nm; Purity: 99.9%.	Marin diatom *Phaeodactylum tricornutum*	Algal cultures were supplied with NPs (0–80 μM) for 48–96 h.	Algal growth; photosynthetic pigment content	Algal growth: slightly stimulated by 48 h of 10 μM Cu-NPs and inhibited by the same dose for 96 h. Greater concentrations (20, 40, 80 μM) significantly inhibited algal growth in a time dependent manner. Photosynthetic pigment content: slightly increased by 10 μM and decreased by 40 μM Cu-NPs (by 41.4%, 47.2%, 44.3% and 76.6%, 83.6%, and 76.1% for chlorophyll a, c and carotenoids, after 48 and 96 h, respectively).	Zhu et al. [23]
Cu- and Se-carboxylated with citric acid nanoaquachelates	Size: ~100 nm.	Green algae *Chlorella vulgaris*	Algal cultures were supplied with NPs (0.67–40 μg/mL) for 6–24 days.	Algal growth: biomass production	Increased by 0.67–4 μg/mL of Cu-nanocarboxylates (~20% biomass increase); inhibited by 20 to 40 μg/mL after the 12th day of cultivation. Decreased by 0.07 and 0.2 μg/mL of Se-nanocarboxylates (6 and 12 days of culture); stimulated by 0.4–4 μg/mL (40–45% biomass increase).	Mykhaylenko and Zolotareva, [24]
**Plants and seedlings**						
MW-CNTs	Size: 6–9 nm large, 5 μm length; Purity: 95%.	*Zea mays* seedlings	Agar culture medium containing MW-CNTs (0–60 μg/mL) was used for seed growth (7 days)	Plant indices of growth and water absorption	Water content (shoots): increased at 10 μg/mL, decreased at higher concentrations. Dry weight (seedlings, roots, shoots): increased at 20 μg/mL, decreased thereafter.	Tiwari et al. [25]
PEG-coated and carbon-coated Ag-NPs	Size: 5 and 10 (PEG-Ag-NPs); 25 nm (carbon coated Ag-NPs).	Poplars (*Populus deltoides* × *nigra*) and *Arabidopsis thaliana*	Plants were exposed to 0–100 μg/mL NPs.	Growth parameters	Evotranspiration (poplar): enhanced by 1 μg/mL carbon-Ag-NPs (42% vs. controls) and by 0.1 μg/mL PEG-Ag-NPs (43% vs. controls); decreased by 100 μg/mL carbon-Ag-NPs (87%). Root and stem biomass (poplar): increased by 63% and 46% after exposure to 1 μg/mL for 11 days carbon-Ag-NPs. Fresh weights of roots, stem and leaves (poplar): increased by 0.1 μg/mL PEG-Ag-NPs (48%, 50%, 39% vs. controls); reduced by 100 μg/mL carbon Ag-NPs (87%, 42%%, 81%). Growth (*Arabidopsis*): enhanced (more extensively expanded leaves) by 1 μg/mL, and inhibited by 100 μg/mL carbon-Ag-NPs. Root growth and shoot weights were increased by 0.01 and 0.05 μg/mL of 5 nm or 0.01 and 0.02 μg/mL fo 10 nm PEG-Ag-NPs and inhibited by 1 μg/mL of these NPs.	Wang et al. [26]
Ag-NPs	Shape: spherical; Size: 10–30 nm.	Common bean (*Phaseolus vulgaris*) and corn (*Zea mays*) plants	Ag-NPs (15 mL at 0–100 μg/mL) were daily supplied to plants for 12 days.	Plant growth parameters	Shoot and root length; fresh and dry weight; leaf area; chlorophyll and carbohydrate content: increased with concentrations up to 60 μg/mL, decreased by higher, 80 and 100 μg/mL.	Salama et al. [27]
Ag-NPs	Shape: spherical; Size: 35 ± 15 nm; Surface functionalization: PVP.	Vanilla shoots (2 cm long)	Shoots were exposed to 0–200 μg/mL NPs for 30 days of culture in a recipient for automated temporary immersion.	Shoot multiplication and length	Number of shoot per explant: 25 and 50 μg/mL NPs: 14.33 and 14.89 respectively; 200 μg/mL: 4.55. Shoot length: 25 and 50 μg/mL NP: 14.33 and 14.89 cm, respectively; 100 and 200 μg/mL: 1.14 ± 0.07 and 0.82 ± 0.6 cm, respectively.	Spinoso-Castillo et al. [28]
PVP coated Ag-NPs	Shape: spherical; Size: 35 ± 15 nm.	Sugarcane shoots	Shoots were placed in a temporary immersion bioreactor in which NP solutions were added (0–250 μg/mL) for 30 days.	Shoot multiplication rate and length	The treatment with 50 and 100 mg/L Ag-NPs induced the greatest shoot number (increased by 35% and 28%, respectively) and length (increased by 52% and 48%, respectively). The lowest Ag-NP concentration (25 μg/mL) had no effect on the evaluated variables. The highest Ag-NP concentration (250 μg/mL) caused a reduction in shoot number and length.	Bello-Bello et al. [29]
Ag-NPs	Size: 10–20 nm.	Seedlings of wheat variety Narc-2009 (*n* = 10 per pot)	Pot soil was soaked with Ag-NPs (0–150 μg/mL) solution or distilled water in control treatment.	Seedling growth; yield attributes and nutrient use efficiency	Ag-NPs at 25 and 50 μg/mL significantly improved maximum leaf area (19.7 and 18.18 cm^2^, respectively, vs. 15 cm^2^ in controls); grain yield (*n* = 29 grains per spike at 25 μg/mL); and nutrient use efficiency (N: 74.3%; K: 89.0%; P: 72.5% at 25 μg/mL). Greater concentrations decreased leaf area, grain yielded (*n* = 11.5 grains per spike at 150 μg/mL) and nutrient use efficiency (N: 36.4%; K: 67.88%; P: 41.3% at 150 μg/mL).	Jhanzab et al. [30]
Ag-NPs	Size: 10–20 nm.	Seedlings of wheat variety Narc-2009 (*n* = 10 per pot)	Seedlings were applied Ag-NPs (0–150 μg/mL) through a blended Murashige and Skoog medium.	Germination, seedling growth, yield attributes	Germination (medium): Ag-NPs had no effect (25–75 μg/mL), and reduced germination (100–150 μg/mL) compared to controls. Number of seminal roots (medium): increased by 25–75 μg/mL, decreased by 100–150 μg/mL, compared to controls. Growth parameters (medium): higher leaf area, fresh weight, dry weight and root biomass was observed with 25 μg/mL Ag-NPs as compared to control after one week of growth in nutrient medium. Growth parameters (NP added soil): more number of grains per spike was produced with 25 and 50 μg/mL, while a decrease was observed with 75–100 μg/mL compared to controls; grain yield per pot was higher with 25 μg/mL, maximum reduction was evident with 150 μg/mL compared to controls.	Razzaq et al. [31]
Metal nanoform colloidal solution	Metal NPs in solution (size in nm; dose in μg/mL): Ag (30–50; 150); Cu (100–150; 200); Fe (20–30; 300); Zn (30–50; 150); Mn (20–30; 150).	Soybean seeds and plants	Pre-sowing seed treatment with metal colloids at 120 or 240 μg/mL; pre-sowing (120 μg/mL) combined with vegetative treatment.	Oxidative stress; lipid peroxidation	Pre-sowing treatment with 120 μg/mL nanosolution increased (12%) lipid peroxidation, while treatment with 240 μg/mL and 120 μg/mL + vegetative treatment decreased the oxidative process (19% and 10%, respectively).	Taran et al. [32]
**Nematodes and larvae**						
Ag-NPs	Size: 8 nm.	*Caenorhabditis elegans* nematodes	Nematodes (*n* = 6) were exposed to 0–4 μg/mL NPs for 72 h.	Reproduction outcome: number of offspring	Short-term test: mean number of offspring (89 and 117, at the 1st and 2nd test, respectively) at 0.0625 μg/mL was higher compared to controls (75) (n.s.). Mean number of offspring at higher doses (2, 4 μg/mL) was significantly reduced compared to controls. Full life-cycle brood size (mean): 182 controls compared to 168, 98 and 5 juveniles at 0.0625, 0.7 and 2.1 μg/mL. No eggs were laid by nematodes exposed to 6.5 μg/mL.	Tyne et al. [33]
CeO_2_-NPs	CeO_2_-NPs (1) Shape: spherical; Size: 2–5 nm; Surface functionalization: tri ammonium citrate layer; Uncoated CeO_2_- spherical NPs (2) Size: 2–5 nm; Uncoated CeO_2_-nano-plates (3) Size: 10–60 nm.	Microcosms containing microbial communities, diatoms and chironomid larvae.	Fresh NP suspensions (50 mL of 93.4 mg/L) were added 12 times over 4 weeks to obtain the final concentration of 1 mg/L. Chironomid larvae were added after 1 week of contamination.	Larval growth and teratogenicity	The teratogenicity induced by CeO_2_-NPs (1) (frequency and seriousness of deformities) was significantly less than that in the control conditions. CeO_2_-NPs (2) and (3) conditions did not show differences compared to controls, although a larger number of larvae lacking one or more teeth was observed with CeO_2_-NPs (2).	Bour et al. [34]
**Superior aquatic organisms**						
ZnO-NPs	Size: 40–100 nm; Surface area: 10–25 m^2^/g.	*Xenopus laevis* embryos (*n* = 15 per dose, per replicate)	Tadpoles were exposed to aqueous suspensions beginning in ovo through metamorphosis (0–2 μg/mL).	Developmental assessment	SVL: on days 10–20, 0.067 μg/mL had significantly longer SVL than controls. On days 10–46, 0.305, 0.513 and 0.799 μg/mL tadpoles were shorter than controls. Total body length: decreased from day 10 to 46, by 0.305, 0.513 and 0.799 μg/mL ZnO-NPs; increased, on days 10, 15, and 35, by 0.067 μg/mL ZnO-NPs compared to controls. Hind limb length: decreased by 0.305, 0.513 and 0.799 μg/mL from day 10 to 46, increased, from day 20 to 35, by 0.067 μg/mL ZnO-NPs compared to controls.	Nations et al. [35]
ZnO-NPs	Size: 40–100 nm; Surface area: 10–25 m^2^/g.	*Xenopus laevis* embryos (*n* = 10 per dose, per replicate)	Nominal solutions of ZnO-NPs (0.1–31.6 μg/mL) were employed for a subacute 96 h exposure protocol for daily solution exchange.	Total body length	Increased by 0.1, 0.316, and 1 μg/mL ZnO; decreased by 10 and 31 μg/mL.	Nations et al. [36]
CuO-NPs	Size: 23–37 nm; Surface area: 25–40 m^2^/g.	*Xenopus laevis* embryos (*n* = 15 per dose, per replicate)	Nominal solutions of CuO-NPs (0.01875–2.5 μg/mL) were employed for 14 and 47 days.	Total body length	All tadpoles in solutions containing less than 0.15 μg/mL CuO-NPs achieved significantly longer total body length than controls in chronic treatment. Tadpoles in the 0.3 μg/mL had lower total body length than controls in chronic treatment.	Nations et al. [37]
Ag-NPs	Size: 5 nm.	Lessepsian-entry bivalve sea mussel *B. pharaonis*	Animals were treated with 2–40 μg/L NPs for 8 days.	Respiration and heartbeat rate	Overall (8 days) respiration rate: 2 and 40 μg/L increased the rate, while 20 μg/L did not show differences compared to controls. RR dynamics during the 8-day period of exposure: a fair increase followed by acclimation to control values was induced by 40 μg/L. A delayed RR intensification was seen with 2 μg/L NPs.	Saggese et al. [38]
**Rats**						
Pd-NPs	Shape: spherical; Size: 10 nm; Size distribution: 4–16 nm.	20 female pathogen-free Wistar rats	Single intravenous injection (via the tail vein) of vehicle (control group) and 0.012, 0.12, 1.2 and 12 μg/kg Pd-NPs (exposed rats).	Production and release of different cytokines (IL-1α, IL-2, IL-4, IL-6, IL-10, IL-12, GM-CSF, INF-γ and TNF-α)	Exposure to Pd-NPs was able to affect immune response since the mean serum concentrations of all cytokines decreased after the administration of 0.012 μg/kg Pd-NPs, while their levels exceeded the control values at higher doses of exposure (0.12, 1.2 and 12 μg/kg).	Iavicoli et al. [39]

Ag-NPs, silver nanoparticles; CeO_2_-NPs, cerium oxide nanoparticles; Cu-NPs, copper nanoparticles; Fe, iron; GM-CSF, Granulocyte macrophage- colony stimulating factor; IL, interleukin; INF-γ, interferon-γ; K, potassium; MIC, minimum inhibitory concentrations; Mn, manganese; MW-CNTs, multi-walled carbon nanotubes; N, nitrogen; P, phosphorus; Pd-NPs, Palladium Nanoparticles; PEG, polyethylene-glycol; PVP, Polyvinylpyrrolidone; RR, respiration rate; SVL, Snout vent length; QD, quantum dot; TNF, tumor necrosis factor; ZnO-NPs, zinc oxide nanoparticles.

## Abbreviations

A549	Human lung adenocarcinoma cells
Ag-NPs	Silver Nanoparticles
AgNO_3_	Silver nitrate
Caco2	Human epithelial colorectal adenocarcinoma cells
CeO_2_-NPs	Cerium oxide nanoparticles
Cu-NPs	Copper Nanoparticles
ENM	Engineered Nanomaterial
Fe	Iron
FSF1	Normal diploid human facial skin fibroblasts
HepG2	Human hepatoma derived cell line
GM-CSF	Granulocyte macrophage- colony stimulating factor
K	Potassium
IL	Interleukin
INF-γ	Interferon-γ
MIC	Minimum Inhibitory Concentrations
Mn	Manganese
MW-CNTs	Multi-Walled Carbon Nanotubes
N	Nitrogen
N_2_O	Nitrous oxide
NDs	Nano Diamonds
NPs	Nanoparticles
n.s.	Not significant
P	Phosphorous
Pd-NPs	Palladium Nanoparticles
PEG	Polyethylene-Glycol
PVP	Polyvinylpyrrolidone
QD	Quantum Dot
RR	Respiration Rate
SiO_2_-NPs	Silica Nanoparticles
SVL	Snout Vent Length
TNF-α	Tumor necrosis factor-α
ZnO-NPs	Zinc Oxide Nanoparticles
